# Chasing the Ghost Proteome in the Dark Matter

**DOI:** 10.1016/j.mcpro.2025.101076

**Published:** 2025-09-29

**Authors:** Tristan Cardon, Isabelle Fournier, Michel Salzet

**Affiliations:** 1 Univ. Lille, Inserm, CHU Lille, U1192 - Protéomique Réponse Inflammatoire Spectrométrie de Masse - PRISM, Lille, France; 2 Institut Universitaire de France, Ministère de l'Enseignement supérieur, de la Recherche et de l’Innovation, 1 rue Descartes, Paris, France

**Keywords:** proteogenomics, small encoded peptides, ghost proteome, dark antigen, biological function

## Abstract

Emerging evidence shows that translation from noncanonical ORFs produces a diverse set of biologically active proteins. These ORFs reside in 5′ and 3′ UTRs, long noncoding RNAs, overlapping frames within annotated genes (dual coding), or pseudogenes and can initiate at non-AUG start codons. The resulting products, variously termed microproteins, small proteins, small ORF–encoded peptides, and alternative proteins, modulate fundamental cellular processes, including metabolic flux and epigenetic regulation. We consolidate these entities under the umbrella of the ghost proteome, a functional proteome arising from the genome’s presumed “dark matter.” This concept is distinct from the dark proteome, which refers to regions of canonical proteins lacking structural, functional, or experimental annotation and is not necessarily derived from noncanonical loci. Recognizing the ghost proteome expands the boundary of what is considered protein coding, demands harmonized nomenclature and database integration, and motivates systematic discovery and functional characterization. By reframing sequences once dismissed as noncoding or “junk,” the ghost proteome compels a re-evaluation of genome annotation and reveals new opportunities to interrogate biology and disease.

## Highlights

• We define the ghost proteome and distinguish it from the dark proteome.
• We map noncanonical ORFs and their products across RNAs contexts.
• We integrate Ribo-Seq with stringent proteogenomics and machine-learning rescoring.
• We reveal links to disease and targets in cancer, metabolism, and neuroimmune axes.

## Graphical Abstract

This diagram illustrates the concept of the "Ghost proteome," which encompasses proteins and peptides that are difficult to detect or are translated from noncanonical parts of the genome. This group includes alternative proteins translated from alternative ORFs, which contain the subset of small ORF (smORF)–encoded peptides produced from smORFs. The ghost proteome also features small proteins, a size-defined category that, along with smORF-encoded peptides and other noncanonical products like those from stop-codon readthrough, shows significant overlap and interrelation, as indicated by the dashed arrows. Finally, the dark proteome represents proteins that are predicted from genomic sequences but have not yet been experimentally observed.

**Figure:**
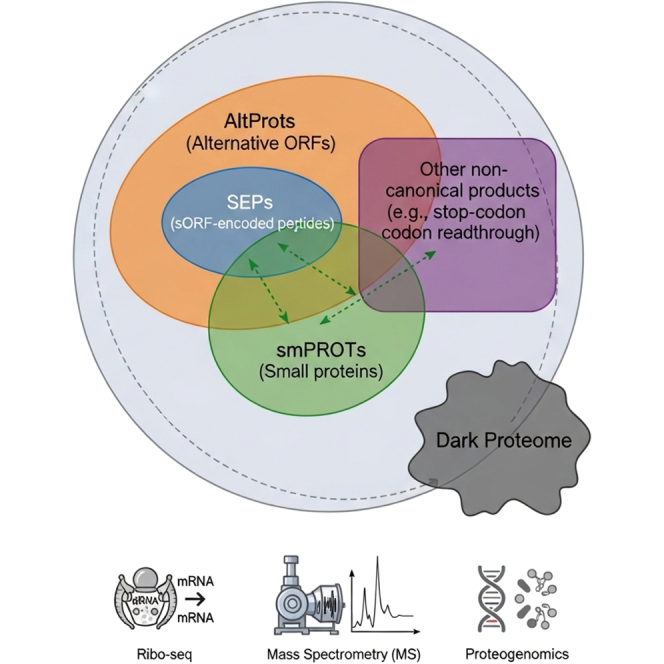

## In Brief

Non-canonical proteins arising from sequences previously presumed noncoding, such as 5′/3′ UTRs, alternative reading frames of mRNAs, pseudogenes, or long noncoding RNAs generate a functional “ghost proteome” validated by Ribo-seq/proteogenomics, distinct from the “dark proteome,” with translational impact in cancer, metabolism and neuro-immune biology.

**Fig. 1:**
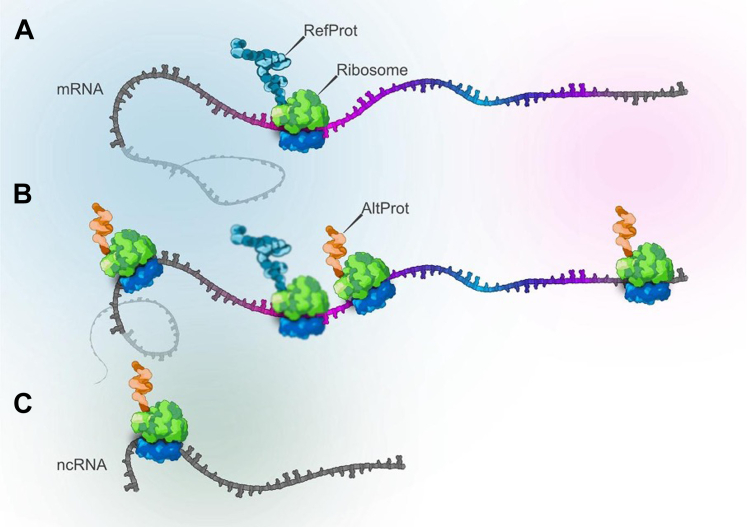
**Schematic representation of noncanonical translation initiation sites.***A,* in the conventional view of protein translation, a ribosome initiates translation at a canonical start codon within the coding sequence (CDS) of an mRNA, flanked by UTRs (5′ and 3' UTRs, shown in *gray*). This results in the synthesis of a reference protein (RefProt, shown in *blue*). *B,* however, alternative translation initiation sites can occur outside the annotated CDS. Ribosomes may initiate translation within the 5′UTR, 3′UTR, or in alternative reading frames within the CDS itself, leading to the production of alternative proteins (AltProts, shown in *orange*) with amino acid sequences distinct from the RefProt. *C,* in addition, AltProts can also be translated from noncoding RNAs (ncRNAs), expanding the landscape of the translatome beyond the conventional protein-coding gene annotations.

For decades, mainstream gene annotation efforts focused on ORFs larger than 300 nucleotides, effectively excluding short coding regions below this threshold (1). Although observations of small proteins (smPROTs) or peptides in bacteria and bacteriophages suggested that such entities might exist (2), it was widely assumed that eukaryotic genomes produced negligible amounts of short, functional proteins. This conservative stance changed dramatically with the advent of large-scale proteomic analyses revealing that 10% to 15% of proteins identified were not annotated in the human genome’s predicted proteome but instead could originate from 5′ or 3′ UTRs or shift in +1 or +2 in the coding domain sequence (CDS) of mRNAs or from noncoding RNAs (3, 4, 5, 6) (Fig. 1).

Similarly, the advent of next-generation sequencing and the introduction of ribosome profiling (Ribo-Seq), which sequences ribosome-protected mRNA fragments at subcodon resolution to pinpoint sites of active translation (7, 8, 9), showed that transcripts’ noncoding regions can actually harbor coding information for previously unannotated proteins, confirming their expression in cells. Ribo-Seq unexpectedly revealed widespread translation in regions long thought to be noncoding, unmasking a diverse repertoire of alternative ORFs (AltORFs); interestingly, they are mainly small ORFs (smORFs). Some of these short reading frames reside in the 5′ and 3′ UTRs of mRNAs, also appear in introns, long noncoding RNAs (lncRNAs) (10), and even overlap canonical protein CDSs out of frame with the main CDS (3) (Fig. 1). By revealing these once-discarded regions as potential protein-coding elements, bottom–up proteomic studies (3, 11, 12, 13) and top–down proteomic approaches (intact protein analysis) (14, 15), together with ribosome profiling data (8, 16, 17, 18), have challenged the scientific community to revisit a fundamental question: What constitutes a CDS?

The accumulated evidence soon established that alternative reading frames encode biologically functional products. These products can even come from pseudogenes and non-AUG–initiated ORFs. These products have been given various names because of a lack of consensus in the community, including “microproteins,” “smPROTs,” “small-encoded peptides,” and “alternative proteins” (AltProts) (Table 1). While debates persist and each term carries slight distinctions and dedicated databases, all refer to the same phenomenon: proteins that are not referenced in classical databases and originate from noncanonical regions of transcripts.

**Table 1:** Definition of smPROTs, SPES, AltProts, and noncanonical proteins

Dimension	SmPROTs	SEPs	AltProts	All noncanonical proteins
Primary definition	Any smPROT (typically <100–150 aa), independent of genomic origin	Proteins translated from sORFs/smORFs, usually outside the annotated CDS	Proteins translated from AltORFs relative to the annotated CDS (overlapping or distinct)	Umbrella: proteins not produced from reference canonical CDSs (includes SEPs, AltProts, and other nonstandard translation products)
Key inclusion criterion	Length-based (amino-acid count)	Source-based: translation from a short ORF	Reading-frame/ORF-based: not the reference CDS (AltORF)	Annotation-based: not encoded by the reference CDS set (GENCODE/UniProt)
Typical size	20–150 aa (often 30–120)	10–100 aa (occasionally up to ∼150)	Wide range; many 30–200 aa but can be smaller or larger	Any size (micro to >300 aa)
Can exceed 150 aa?	No (by definition)	Rarely; generally no	Yes, possible	Yes
Reading frame *versus* canonical CDS	Any (same or alternative frame); size is the only constraint	Independent short ORFs (uORFs/dORFs, lncRNA ORFs); may overlap but are not the reference CDS	Alternative frame (+1/+2/−1) or distinct ORF in the same transcript or in noncoding transcripts	Any: alternative frames, distinct ORFs, readthrough, frameshift-derived
Typical genomic sources	Canonical CDS (short proteins), uORFs/dORFs, lncRNAs, pseudogenes, circRNAs	5′-UTR (uORFs), 3′-UTR (dORFs), lncRNAs, pseudogenes, introns, circRNAs, mitochondrial RNAs	Overlapping CDSs, 5′/3′-UTR AltORFs, alternative splice isoforms, lncRNAs, pseudogenes	UTRs, overlapping CDSs, lncRNAs, pseudogenes, introns/retained exons, circRNAs, repeats/transposons; non-AUG start; stop-codon readthrough
Typical transcript types	mRNAs; some from lncRNAs/circRNAs/pseudogenes	lncRNAs; UTRs of mRNAs; pseudogenes; circRNAs	mRNAs (AltORFs); lncRNAs; pseudogenes	mRNAs, lncRNAs, pseudogenes, circRNAs, viral-like/repetitive elements
Examples	NoBody (∼68 aa), Humanin (∼24 aa), BRAWNIN (∼71 aa)	SPAAR (∼90 aa), ASNSD1-uORF microprotein, Humanin (∼24 aa)	AltMiD51 (∼70 aa), AltDDIT3 (∼50 aa), alt-RPL36 (∼34 aa)	AltProts (*e.g.*, AltMiD51), SEPs (*e.g.*, SPAAR), circRNA-encoded peptides (*e.g.*, circZNF609 peptide)
Main detection methods	Ribo-Seq; shotgun/targeted MS; N-terminomics	Ribo-Seq (ORF-quant); MS with custom proteogenomic DBs; peptidomics	Ribo-Seq; proteogenomics (OpenProt); targeted MS; cross-linking MS	Integrated Ribo-Seq + MS; initiation-site mapping (QTI-Seq/TIS-Seq); immunopeptidomics
Common databases/resources	smProt, sORFs.org	sORFs.org, SmProt, RiboCode/PRICE outputs	OpenProt, AltORFev	OpenProt, sORFs.org, SmProt, uORFdb, circRNADb (coding entries)
Typical annotation status (UniProt/RefSeq)	Mixed: some annotated; many newly added or pending	Mostly unannotated historically; growing inclusion	Largely unannotated historically; curated in OpenProt; selected entries emerging in UniProt	Mostly absent historically; inclusion varies by resource and evidence
Relation to the ghost proteome	Only the subset arising from noncanonical ORFs belongs to the ghost proteome	Core component of the ghost proteome	Core component of the ghost proteome	≈ Overlaps strongly with the ghost proteome as commonly used

**Fig. 4:**
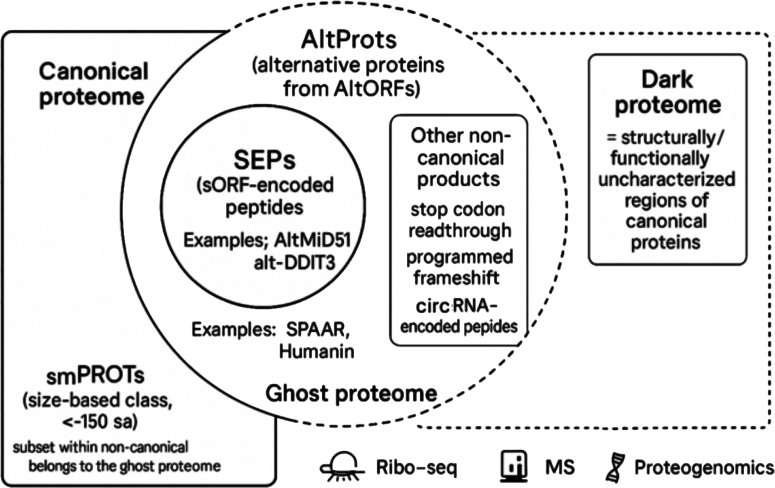
**The *circle* contour marks the ghost proteome (noncanonical translational products).** Within it, the alternative protein (AltProt) ellipse represents proteins translated from alternative ORFs (AltORFs) residing in overlapping frames of annotated genes, UTRs, long noncoding RNAs (lncRNAs), pseudogenes, or alternative transcripts. The *circle* inside AltProts depicts SEPs (sORF-encoded peptides), a subset of AltProts arising from small ORFs (smORFs) (*e.g.*, SPAAR, Humanin); examples of AltProts include AltMiD51 and alt-DDIT3. The *rectangle* denotes other noncanonical products such as stop-codon readthrough, programmed frameshifting, and circRNA-encoded peptides. The small proteins (smPROTs) (∼≤150 aa) correspond to proteins with a size-defined class that crosscuts canonical and noncanonical proteomes; only the noncanonical fraction belongs to the ghost proteome. The dark proteome corresponds to structurally/functionally uncharacterized regions of canonical proteins; this concept is distinct from the ghost proteome. Icons at the *bottom* summarize principal detection/validation modalities: Ribo-Seq, mass spectrometry (MS), and proteogenomics. Set relations: SEPs ⊂ AltProts ⊂ ghost proteome; smPROTs span canonical + noncanonical; dark proteome ⟂ ghost proteome (orthogonal concept).

These noncanonical proteins can modulate multiple cellular processes, from metabolic flux to epigenetic regulation (19, 20, 21, 22). This wealth of unexpected protein diversity constitutes what we call the ghost proteome, not to be confused with the “dark proteome,” which refers to the regions of known protein-coding genes or full-length proteins that lack functional, structural, or experimental annotation (6, 23, 24, 25). Thus, the ghost proteome is derived from the “dark matter” of the genome, but it goes further: it transforms what was once considered noncoding, nonfunctional, or “junk DNA” into a functional, alternative proteome, whereas the dark proteome is not directly derived from the dark matter of the genome since these proteins are canonical, not from the supposed “noncoding” or “junk” DNA (Fig. 4). Thus, it is possible to chase the ghost proteome in the dark matter of the genome.

The early success of Ribo-Seq in identifying noncanonical proteins was complemented and validated by proteogenomic approaches, in which mass spectrometry (MS) data are matched against customized protein databases containing newly predicted noncanonical sequences (such as those cataloged in HAltORF (26) and OpenProt (27)). High-resolution MS instruments began to reveal peptides corresponding to short sequences never cataloged in canonical repositories such as UniProt. To increase confidence in these findings, stringent false discovery rate (FDR) criteria and multipeptide validation were implemented, conclusively demonstrating that numerous predicted AltORFs were indeed translated into functional noncanonical proteins. However, controlling the FDR in proteogenomics remains particularly challenging because of the increased search space generated by large custom databases that include AltORFs. This issue parallels similar challenges in metaproteomics, where vast and complex sequence databases significantly elevate the risk of random matches.

In recent years, advanced computational strategies have emerged to address this limitation. Notably, rescoring approaches such as MS2Rescore (28), which integrate predicted fragmentation patterns and retention times through machine learning models like Prosit (29) or MS2PIP (30), have shown great promise in refining peptide-spectrum match validation. These tools offer improved sensitivity without compromising specificity, recovering true positives that might otherwise be discarded by more conservative classifiers such as Percolator. While Percolator (31) has long been the standard for FDR estimation using semisupervised learning, its stringent filtering in large search spaces can result in the loss of biologically relevant identifications. Rescoring tools now allow for more nuanced control and are increasingly adopted in routine workflows (25, 32, 33). Additional studies have used integrative pipelines combining Ribo-Seq data, evolutionary conservation metrics, and targeted proteomics to filter out spurious signals arising from ribosome pausing or scanning (34). These integrative methods have also revealed the surprising breadth of mechanisms by which AltORFs arise (6, 35). Some AltORFs overlap a canonical CDS in a different frame, thereby doubling the protein-coding capacity of a single transcript. This so-called “polycistronic” ability of eukaryotic cells, traditionally considered monocistronic, is nonetheless well accepted in the context of viral infections. A virus introduces its RNA into a host cell, allowing translation of multiple viral proteins from a single mRNA. Other AltORFs use non-AUG start codons, such as CUG or GUG, which expand the translational landscape under specific cellular or stress conditions (36, 37). The result is a remarkable enrichment of potential protein-coding elements far beyond the conventional gene catalog, as evidenced by the enormous diversity of noncanonical proteins now confidently detected across a range of eukaryotes (4, 5, 11, 19, 38).

## Mechanisms of Alternative Translation and Their Regulatory Complexity

As researchers have delved deeper into the origin of these hidden proteins, it has become clear that canonical AUG-initiated translation represents only a fraction of the dynamic processes contributing to the ghost proteome. Some noncanonical proteins originate from upstream ORFs in the 5′ UTR, which have historically been viewed solely as regulatory sequences that prevent translation of downstream coding regions (39). In many cases, these upstream ORFs encode smPROTs with discrete biological effects, including metabolic modulation and stress responses. Other noncanonical proteins arise from downstream ORFs in the 3′ UTR, demonstrating that almost any region of a transcript can become functionally relevant given a permissive context and initiation signals (40). Meanwhile, overlapping ORFs generate proteins with a completely different sequence, which can either complement or antagonize the function of a canonical protein. This overlap can facilitate coherent regulation when both products are involved in related pathways, as seen with certain lncRNA-derived proteins that modulate the activity of oncogenic factors in cancer cells (6, 41). While AUG remains the dominant start site under basal conditions, stress responses or tissue-specific factors can increase initiation at alternative start codons, thereby broadening the adaptive capacity of the proteome (36). This flexibility helps explain why noncanonical proteins are frequently implicated in acute or context-dependent cellular events, ranging from stress responses and metabolic changes to brain disorders and immune reactions (42, 43, 44, 45, 46, 47, 48, 49, 50, 51, 52). By existing in alternative reading frames or using cryptic initiation codons, AltProts confer additional layers of control and can rapidly alter the cellular proteome composition in response to physiological or pathological stimuli (51, 53). The adaptability of these shorter sequences often corresponds to a regulatory or a modulatory role. For example, in leukemia cell lines K562 and MOLT-4, it was demonstrated that four of the 16 noncanonical proteins identified were differentially expressed (54). In addition, a study of the mitochondrial DNA genomic region showed it encodes MOTS-c, a 16-amino acid noncanonical protein that plays a role in muscle and fat metabolism (55) (Table 2). Interestingly, the noncanonical protein EMBOW (Endogenous Microprotein Binder Of WDR5), dually encoded in the human *SCRIB* gene, interacts with WDR5 and regulates its binding to multiple partners, including KMT2A and KIF2A. EMBOW is cell cycle regulated, with two expression peaks at late G1 and G2/M phases. Loss of EMBOW decreases WDR5’s interaction with KIF2A, aberrantly shortens mitotic spindle length, prolongs the G2/M phase, and delays cell proliferation (56).

**Table 2:** Representative noncanonical “ghost” proteins and their contexts

Noncanonical protein (origin)	Transcript/ggene source	Functional annotation	Disease or pathway relevance
AltAKT (alternative AKT microprotein)	*AKT1* mRNA (AltORF within CDS)	Short peptide translated from an overlapping frame; likely modulates AKT pathway activity	Identified in human tumors; implicated in cancer cell proliferation (62)
AltEDARADD (alternative EDARADD protein)	*EDARADD* mRNA (AltORF within CDS)	Microprotein in an alternate frame; may influence NF-κB/death receptor signaling (mechanism under study)	Detected in cancers; potential role in prosurvival/proliferative signaling (11, 62)
HOXB-AS3 peptide (lncRNA-derived)	*HOXB-AS3* lncRNA (HOXB locus)	53-aa peptide binds HNRNPA1; blocks stabilization of oncogenic transcripts	Tumor suppressor in colon cancer; restoration inhibits tumor growth (41)
AltATAD2 (alternative ATAD2 protein)	*ATAD2* mRNA (AltORF overlapping CDS)	Interacts with AUF1 and RPL10; suggests ribosome-associated translational control	Implicated in cancer cell biology *via* altered translation of growth-related mRNAs (11)
EMBOW (Endogenous Microprotein Binder Of WDR5)	*SCRIB* (dual-coding; AltORF)	73-aa protein binds WDR5; modulates interactions with KMT2A and KIF2A; cell-cycle regulated	Controls chromatin/mitosis; loss shortens spindle, prolongs G2/M, slows proliferation (56)
Mitoregulin (LINCMD1 microprotein)	*LINCMD1* (lncRNA)	∼56-aa mitochondrial microprotein; stabilizes respiratory supercomplexes; boosts OXPHOS	Supports metabolic homeostasis; therapeutic potential in obesity/T2D (65, 66, 67)
Myoregulin (regulin family)	*MRLN* (formerly lncRNA)	46-aa SERCA regulator in SR membrane; calcium-handling control (family includes PLN, SLN, DWORF)	Muscle contraction/relaxation; links to heart failure and muscular dystrophy (69, 108)
MOTS-c (mitochondrial peptide)	*mtDNA 12S rRNA* (short ORF)	16-aa peptide; translocates to cytosol/nucleus; activates AMPK; affects folate/purine metabolism	Improves insulin sensitivity; protects against diet-induced obesity; cytoprotective roles (55, 92)
Heimdall (astrocytic AltProt)	ncRNA related to Ig κ light-chain variable region (astrocyte specific)	∼100-aa protein; “gatekeeper” of astrocyte → neuroprogenitor conversion	Neural development/regeneration; potential roles in brain injury/neurodegeneration (49)
T1 (T-cell activation microprotein)	smORF in primary human T cells (detected on activation)	Rapidly induced upon TCR stimulation; provides negative feedback to dampen activation	Tunes immune responses; prevents overactivation (72)

Examples of noncanonical proteins (“ghost proteins”) encoded by small ORFs or AltORFs, illustrating their diverse origins and functions. Each entry highlights how these microproteins integrate into cellular pathways, from cancer and genomic stability to metabolism, muscle physiology, and immune regulation. Notably, many originate from transcripts once believed noncoding (alternative mRNAs, lncRNAs, or even mitochondrial RNA), yet they execute important regulatory roles. These cases underscore the biological relevance of the ghost proteome in health and disease.

AUF1, poly(U)-binding/degradation factor 1; CDS, coding domain sequence; KMT2A, lysine methyltransferase 2A; OXPHOS, oxidative phosphorylation; RPL10, ribosomal protein L10; SERCA, sarco/endoplasmic reticulum Ca^2+^-ATPase; SR, sarcoplasmic reticulum; TCR, T-cell receptor; WDR5, WD repeat–containing protein 5.

Computational advances have also driven structural insights (56). AlphaFold2 (57), which uses deep learning to predict protein structure from sequence, has facilitated the generation of structural hypotheses for numerous noncanonical proteins (58). However, empirical validation remains crucial to confirm how these microproteins fold *in vivo* and to dissect how post-translational modifications, such as phosphorylation, acetylation, and ubiquitination, fine-tune their functions (14, 15). Recently, several works have revealed that microproteins use post-translational modifications to regulate their stability, localization, or interactions, reflecting a complexity comparable to canonical proteins. Cross-linking mass spectrometry (XL-MS) studies have revealed the ability of microproteins to interact with reference (canonical) proteins or with other noncanonical proteins (11, 51, 59, 60, 61).

## Functions in Physiology and Disease

The increasing focus on noncanonical proteins has highlighted their profound impact on cellular homeostasis and disease progression. AltAKT and AltEDARADD, alternative microproteins translated from the AKT1 and EDARADD transcripts, respectively, have been identified in tumors and are implicated in cancer cell proliferation (62). Similarly, the lncRNA-derived protein HOXB-AS3 interacts with the RNA-binding protein HNRNPA1, blocking HNRNPA1’s ability to stabilize certain oncogenic mRNAs and thereby suppressing colon tumorigenesis (41). Another example is AltATAD2, an AltProt from the ATAD2 transcript, which interacts with the poly(U)-binding degradation factor 1 (AUF1) and with ribosomal protein L10 (RPL10), suggesting it localizes to ribosomes and impacts translation (11). Such examples illustrate the ability of these noncanonical proteins to either promote or suppress cancer, depending on the nature of their interaction partners (63, 64).

Noncanonical proteins also have significant impacts on metabolic regulation. Mitoregulin, an smORF-encoded microprotein expressed in mitochondria, supports the formation of mitochondrial respiratory supercomplexes, thereby improving oxidative phosphorylation and reducing metabolic dysfunction (65). This protective role suggests that modulating mitoregulin expression may have therapeutic potential in metabolic diseases such as obesity or type 2 diabetes (66, 67). In parallel, members of the myoregulin family fine-tune calcium handling in muscle cells; for instance, myoregulin (encoded by the *MRLN* gene, formerly misannotated as an lncRNA) is a 46-amino acid peptide that embeds in the sarcoplasmic reticulum membrane and regulates Ca^2+^ uptake by the sarco/endoplasmic reticulum Ca2+-ATPase pump. Myoregulin and its analogs (phospholamban, sarcolipin, endoregulin, DWORF, etc.) form a family of smORF-encoded regulators of calcium signaling in muscle (68, 69). By inhibiting sarco/endoplasmic reticulum Ca2+-ATPase, myoregulin modulates calcium cycling in skeletal muscle, influencing exercise performance and muscle fatigue. Dysregulation of these micropeptides is linked to heart failure and muscular dystrophy (69), making them potential targets for treating cardiac and muscle diseases through adjustment of calcium homeostasis.

Equally intriguing are noncanonical proteins that modulate neuronal and immune functions. For example, certain microproteins interact with synaptic vesicle proteins to regulate neurotransmitter release (70, 71). A recently characterized microprotein called HEIMDALL acts as a gatekeeper of astrocyte-to-neuronal progenitor conversion (49). HEIMDALL is produced from an astrocyte-specific ncRNA with immunoglobulin kappa light chain–like sequences, and its expression in glial cells can induce neuroprogenitor characteristics. This novel factor in neural development and regeneration may play a role in the brain’s injury response or in neurodegenerative disease, and its discovery opens possibilities for regenerative medicine, though its mechanism in controlling cell identity is still being unraveled.

**Fig. 2:**
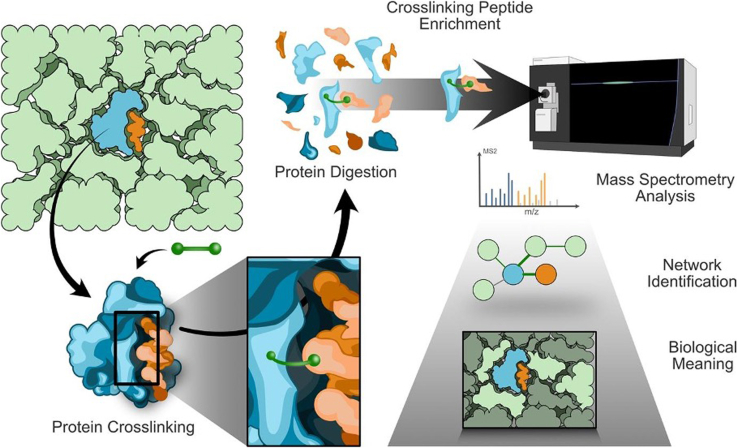
**Overview of the crosslinking mass spectrometry (XL-MS) workflow for the identification of noncanonical protein interactions.** The cellular proteome, composed of both reference proteins (in *blue*) and noncanonical proteins (in *orange*), is stabilized using a chemical crosslinker that covalently links proximal amino acids between interacting proteins. Following crosslinking, proteins are extracted and digested into peptides. Crosslinked peptides, often under-represented, are enriched to enhance their detection and subsequently analyzed by MS. MS data are then processed to identify crosslinked peptides and annotate interaction pairs. The resulting interaction networks are further integrated with publicly available interactomics datasets to reconstruct signaling pathways and infer the potential involvement of AltProts in known biological processes. AltProt, alternative protein.

Moreover, large-scale and unsupervised approaches based on XL-MS combined with shotgun proteomics have been employed to systematically map the functional roles of AltProts by identifying their reference protein interactors (Fig. 2). In this context, Gene Ontology and pathway enrichment analyses revealed that reference proteins interacting with AltProts are involved in cell motility and tRNA regulation (51). Using such an approach along with subcellular fractionation, 12 unique AltProts were identified in a human ovarian epithelial cell line (T740), with 16 crosslinks detected between AltProts and reference proteins (61). Notable interactions included an AltProt (IP_2292176, also known as AltFAM227B) binding to HLA-B and a histone (HIST1H4F) interacting with several noncanonical proteins that may play roles in mRNA transcription (61). In ovarian cancer, a proteogenomic strategy identified 30 AltProts exclusively in SKOV-3 and PEO-4 cell lines. Among them, an AltProt variant (IP_715944) translated from DHX8 was found to carry a mutation (p.Leu44Pro). XL-MS revealed an interaction between POLD3 and another AltProt (IP_183088), and molecular docking placed the AltProt at the POLD3–POLD2 interface, hinting at a potential influence on DNA replication and repair (59). Similarly, proteogenomic profiling of primary human T cells detected 411 novel noncanonical proteins (including 83 newly synthesized “nascent” peptides). Upon T-cell activation (with phorbol 12-myristate 13-acetate/ionomycin or CD3/CD28 stimulation), three noncanonical proteins (dubbed T1, T2, and T3) were shown to modulate T-cell activation, with T1 providing negative feedback to dampen the T-cell response (72).

Thus, these diverse functional roles underscore the broad influence of the ghost proteome on health and disease. To illustrate the range of disease contexts in which smORFs and AltORFs are implicated, Table 1 highlights several representative noncanonical proteins, their genomic source, biological function, and associated pathology. Each example shows how microproteins can integrate into core cellular pathways, from metabolic homeostasis and inflammatory signaling to genomic stability. Their small size often belies a surprisingly potent ability to reprogram or fine-tune cell behavior.

## Evolutionary Perspectives and Rapid Emergence of smORFs

**Fig. 3:**
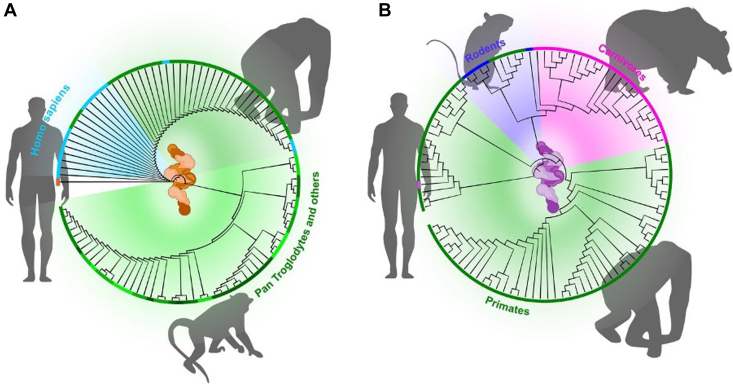
**Phylogenetic analysis of noncanonical coding sequences (CDSs) using BLAST alignment.***A,* the CDS of IP_652563, a noncanonical protein translated from the noncoding transcript ENST00000382641.1, shows strong sequence conservation among primates but no detectable homology outside this clade. This pattern suggests a recent evolutionary origin, potentially limited to bipedal lineages. *B,* in contrast, the CDS for IP_581419, derived from the noncoding transcript ENST00000634439.1, exhibits broader evolutionary conservation. Homologous sequences are observed in rodents and large carnivores, indicating an older origin of this alternative ORF. These observations illustrate the heterogeneous evolutionary trajectories of AltProts, highlighting that while some may represent recent genetic innovations, others have emerged earlier and potentially carry conserved biological functions across diverse mammalian lineages. AltProt, alternative protein.

Analyses of smORFs through an evolutionary lens reveal that some are highly conserved across phyla, suggesting critical ancestral roles (Table 3). For example, small regulatory peptides involved in muscle physiology show evidence of purifying selection in vertebrates (73). Conversely, many smORFs appear to have emerged relatively recently in certain lineages, suggesting a capacity for *de novo* gene birth from sequences once considered noncoding (74, 75). This dynamic interplay between ancient conservation and rapid emergence underscores the evolutionary plasticity of eukaryotic genomes (Fig. 3). Novel smORFs that confer adaptive advantages may be rapidly integrated into existing regulatory networks, whereas others may remain lineage-specific curiosities that can tweak cellular processes under selective pressure (6). The idea that noncanonical proteins can acquire functionality and become essential in specific physiological or pathological contexts adds intrigue to the concept of the dark proteome. It suggests that eukaryotic organisms have more “genomic plasticity” than previously thought, enabling them to evolve new regulatory modules capable of controlling pathways such as apoptosis, immune signaling, or developmental timing.

**Table 3:** Evolutionary classes of smORF-encoding genes in the “ghost proteome”

Conservation level	Approximate evolutionary age	Characteristics and examples	Proposed functional constraints
Lineage specific (*e.g.*, primate specific smORFs)	Very recent (*e.g.*, primate lineage ∼6–10 Mya; some human specific)	No homologs outside the lineage; often arise *de novo* from previously non-CDSs; enriched in primate-specific lncRNAs/UTRs. *Examples:* multiple primate-only lncRNA-encoded peptides (74)	Generally weak purifying selection initially; many are condition specific or transient. A subset acquires beneficial functions (possible positive selection) and becomes constrained within the lineage
Clade-restricted (*e.g.*, mammal/vertebrate conserved)	Moderately ancient (mammals ∼100–200 Mya; vertebrates ∼500 Mya)	Present across a broad clade; retained from an ancestral genome. *Examples:* myoregulin/phospholamban/sarcolipin family across vertebrates; mtDNA-encoded humanin conserved in mammals (69, 91)	Notable purifying selection; sequences conserved because of essential roles (*e.g.*, calcium handling, core metabolism). Disruption may be deleterious
Deeply conserved (across phyla)	Ancient (>500 Mya)	Rare clear cases because of rapid sequence divergence of tiny proteins; sometimes only motifs or positions are conserved; functional analogs exist across distant taxa	If truly crossphyla, extremely strong purifying selection (conserved motifs). Some apparent cases may reflect convergent evolution addressing common functional needs
Intermediate (species group specific)	Intermediate (*e.g.*, eutherian specific ∼50–100 Mya; rodent specific)	Present in a subset of related species, absent elsewhere; can derive from young gene duplicates or mobile elements	Moderate constraint: conserved within the group, implying benefit in specific contexts (*e.g.*, niche adaptations such as hibernation). Not universally essential outside the clade

Evolutionary categories of smORFs and their encoded proteins, illustrating the spectrum from newly emerged (lineage specific) to ancient, conserved microproteins. The “inferred age” of an smORF influences how constrained its sequence is by natural selection. Recent *de novo* microproteins (*e.g.*, human or primate specific) often show rapid evolution and occasionally novel functionality, whereas older microproteins conserved across many species tend to carry out fundamental cellular roles under strong purifying selection. Intermediate cases exist where a microprotein is conserved in a particular lineage (*e.g.*, all mammals), reflecting an important function that arose later in evolution or a clade-specific adaptation. Understanding these evolutionary contexts can guide functional studies: a highly conserved microprotein is likely crucial for basic physiology (and thus a high-priority subject for mechanistic study), whereas a primate-specific microprotein might be involved in more specialized or recently acquired regulatory circuits but could also offer insight into what makes human biology unique.

Conservation patterns guide prioritization: deeply conserved microproteins often underlie core physiology (73), whereas lineage-specific ones may reveal recent regulatory innovations (74, 75). The regulin family illustrates clade-wide conservation tied to calcium signaling (69).

As interest in noncanonical proteins has grown, so have efforts to translate these fundamental discoveries into practical applications. The presence of certain noncanonical proteins on the surface of cancer cells has motivated their exploration as neoantigens for personalized medicine (76). By targeting antigens arising from tumor-specific noncanonical proteins, immunotherapies may achieve superior specificity and fewer off-target effects compared with therapies directed against canonical proteins that are also expressed in healthy tissues. Other lines of research focus on harnessing the regulatory capacity of microproteins in metabolic or inflammatory pathways. For example, a deeper mechanistic understanding of how mitoregulin alters metabolic flux could inspire novel treatments for metabolic syndrome or help inhibit cancer cell growth by interfering with tumor-specific AltORFs (65, 77). Beyond immunotherapy and metabolism, the small size of noncanonical proteins makes them attractive templates for peptidomimetics designed to modulate protein–protein interactions (69). Meanwhile, improved proteomic workflows have begun to incorporate MS imaging of clinical biopsies to pinpoint the spatial distribution of these proteins and how their expression correlates with pathological features (6). These approaches may eventually lead to noncanonical protein-based biomarkers for early detection and stratification of diseases such as ovarian, colon, or breast cancer. In parallel, antisense oligonucleotides, siRNAs, and CRISPR–Cas9 are being considered to target disease-promoting noncanonical proteins (4, 48, 49, 78, 79, 80), providing a new level of precision medicine.

Despite the transformative insights provided by Ribo-Seq and proteogenomics, several hurdles remain. One lingering concern stems from potential false positives: ribosomes can transiently scan transcripts without producing a stable protein product (34). Rigorous validation protocols, including targeted MS, synthetic peptide spiking, and strict criteria requiring multiple unique peptides for protein identification, are therefore essential (81). In addition, sample preparation for proteomics must be optimized to detect low-abundance and rapidly degraded peptides (62, 82, 83, 84). Another challenge is to firmly establish the functional relevance of each newly discovered noncanonical protein, as not all detected peptides may be biologically significant. However, the accumulation of high-quality peptide evidence, evolutionary conservation data, and clear phenotypic outcomes in knockout or overexpression models collectively support the view that a substantial fraction of these short polypeptides serve critical functions (5, 85, 86, 87). As methods continue to evolve, multiomics integration promises to improve our understanding of how noncanonical proteins interface with transcriptional and metabolic networks (38, 88). For instance, combined long-read RNA-Seq or *de novo* Ribo-Seq and proteomics could track the expression of microproteins in different cell populations, revealing specialized or context-dependent functions. At the same time, advanced structural biology and cryo-electron microscopy techniques tailored to smPROT complexes could further elucidate binding interfaces, providing new angles for therapeutic intervention. By combining these state-of-the-art approaches, from multiomics discovery (89) to *in silico* modeling, researchers will not only enlarge the catalog of hidden proteins but also begin to understand their biology at a depth comparable to canonical proteins. In sum, the coming years should see the ghost proteome gradually emerge from the shadows, with mechanistic insights and translational advances converging to integrate these once-elusive proteins into the mainstream of biology and medicine.

Another promising avenue is the link between the noncanonical proteome and aging. Growing evidence suggests that some microproteins act as modulators of longevity and proteostasis (90). Mitochondria-derived peptides such as humanin (91) and MOTS-c have demonstrated cytoprotective effects and are associated with extended healthspan in model organisms and humans (55, 92). Intriguingly, classic lifespan-extending interventions like caloric restriction and rapamycin were recently shown to restore translation of specific noncanonical ORFs in aged mouse muscle, hinting that the ghost proteome may mediate some benefits of these treatments (93). Unraveling how AltProts influence aging processes, from stress resistance to metabolic regulation, could uncover novel biomarkers of aging and new targets to promote healthy longevity.

The interplay between the ghost proteome and the microbiome is another frontier for exploration. The human microbiome itself harbors an enormous “dark” microproteome (94): thousands of smPROTs encoded by gut microbes have only recently been identified (95). These microbial peptides and the host’s own noncanonical proteins may engage in a previously unappreciated molecular crosstalk. It is conceivable that some bacterial microproteins mimic host hormones or modulate immune receptors, whereas certain host micropeptides could influence microbial growth or composition. Investigating these interactions will be challenging but could yield insights into how our commensal microorganisms affect physiology and disease through hidden protein factors. Bridging the fields of microbiome research and the ghost proteome may thus reveal new dimensions of host–microbe symbiosis and potential therapeutic levers for metabolic and inflammatory diseases.

There are also exciting opportunities to harness the noncanonical proteome in synthetic biology and medicine. Due to their small size and regulatory potency, microproteins could be engineered as control elements in cellular circuits or as therapeutic molecules. For instance, tailored expression of a microprotein in engineered T cells might improve their persistence or stress resistance during cell therapy. Gene delivery studies already hint at the therapeutic potential of these peptides: delivering the micropeptide DWORF to heart muscle *via* adeno-associated virus gene therapy enhances calcium handling and ameliorates cardiomyopathy in animal models (96). Likewise, an analog of the mitochondrial peptide MOTS-c has entered clinical trials for metabolic disease, underscoring the translational promise of mining the ghost proteome (92). In the future, we can envision custom-designed microproteins as drugs or as “tunable knobs” inside cell-based therapies, offering a novel strategy to modulate cellular functions with minimal genetic payload.

Finally, continued innovation in discovery technologies will be pivotal to fully illuminate the ghost proteome. Advanced ribosome profiling techniques, including specialized protocols for organelle-specific translation, are expanding our view of which purportedly “noncoding” sequences produce peptides (19, 43). Improvements in MS now enable detection of ever more peptides in the low-kilodalton range, though challenges remain in capturing very hydrophobic or transient microproteins (6, 60, 61). Complementary computational pipelines are also maturing: for example, machine-learning models can integrate ribosome occupancy data with evolutionary and sequence features to predict which novel peptides are likely to be stable and functional (97, 98). Meanwhile, artificial intelligence–based structure prediction tools can provide initial hints of biochemical function even before experimental characterization. By combining these approaches, multiomics discovery to *in silico* modeling—researchers will not only enlarge the catalog of hidden proteins but also gain detailed insights into their biology. It is increasingly likely that the ghost proteome will be systematically mapped and functionally characterized in the coming years, shedding light on its contributions to both fundamental biology and medicine.

## Conclusion

The concept of a “ghost proteome,” once a fringe notion, is now firmly established in modern molecular biology. Technologies such as ribosome profiling have revealed widespread translation in sequences previously deemed noncoding, whereas high-resolution MS and integrative proteogenomics have validated the existence of hundreds, if not thousands, of functional noncanonical proteins. It is also increasingly clear that intron retention (99), frameshifted internal ORFs (100), and alternative splicing (101) can generate novel microproteins. Intron retention, a form of alternative splicing long neglected in mammalian systems, has been shown to play pivotal roles in normal and disease biology (102). In fact, some retained introns produce neoantigens in tumors (103, 104). Alternative splicing is likewise a known source of neoantigens (105), and immunopeptidomics approaches are rapidly developing to identify such tumor-specific peptides as potential targets (106, 107). These discoveries challenge long-held assumptions about gene structure, expand our understanding of regulatory diversity, and offer exciting prospects for clinical innovation.

Noncanonical proteins derived from smORFs and AltORFs have demonstrated remarkable versatility in orchestrating cellular activities, as evidenced by their roles in muscle physiology, cancer metabolism, mitochondrial function, and inflammatory signaling. Their functions often depend on compact but sophisticated structural motifs, post-translational modifications, and context-specific translation initiation. These proteins also exhibit a fascinating mix of deep conservation and *de novo* emergence, shedding light on the evolutionary plasticity of eukaryotic genomes. While technical and conceptual hurdles remain, particularly regarding rigorous annotation, detection, and functional validation, the ever-improving repertoire of multiomics tools offers a promising path forward. Future research that systematically tests the physiological effects of noncanonical proteins, resolves their structures in detail, and therapeutically targets their functions could usher in a new era of precision medicine. Ultimately, unraveling the “ghost proteome” will deepen our fundamental understanding of biology and may unlock unconventional strategies to diagnose, prevent, and treat a range of diseases that have thus far eluded traditional approaches.

## Conflict of Interest

The authors declare that they have no conflicts of interest with the contents of this article.

## References

[1] Clamp M., Fry B., Kamal M., Xie X., Cuff J., Lin M.F. (2007). Distinguishing protein-coding and noncoding genes in the human genome. Proc. Natl. Acad. Sci. U. S. A..

[2] Sabath N., Wagner A., Karlin D. (2012). Evolution of viral proteins originated de novo by overprinting. Mol. Biol. Evol..

[3] Vanderperre B., Lucier J.-F., Bissonnette C., Motard J., Tremblay G., Vanderperre S. (2013). Direct detection of alternative open reading frames translation products in human significantly expands the proteome. PLoS One.

[4] Delcourt V., Staskevicius A., Salzet M., Fournier I., Roucou X. (2018). Small proteins encoded by unannotated ORFs are rising stars of the proteome, confirming shortcomings in genome annotations and Current vision of an mRNA. Proteomics.

[5] Samandi S., Roy A.V., Delcourt V., Lucier J.-F., Gagnon J., Beaudoin M.C. (2017). Deep transcriptome annotation enables the discovery and functional characterization of cryptic small proteins. eLife.

[6] Cardon T., Fournier I., Salzet M. (2021). Shedding light on the ghost proteome. Trends Biochem. Sci..

[7] Ingolia N.T., Ghaemmaghami S., Newman J.R., Weissman J.S. (2009). Genome-wide analysis *in vivo* of translation with nucleotide resolution using ribosome profiling. Science.

[8] Ingolia N.T., Hussmann J.A., Weissman J.S. (2019). Ribosome profiling: global views of translation. Cold Spring Harb. Perspect. Biol..

[9] Ingolia N.T. (2014). Ribosome profiling: new views of translation, from single codons to genome scale. Nat. Rev. Genet..

[10] Couso J.-P., Patraquim P. (2017). Classification and function of small open reading frames. Nat. Rev. Mol. Cell Biol..

[11] Cardon T., Salzet M., Franck J., Fournier I. (2019). Nuclei of HeLa cells interactomes unravel a network of ghost proteins involved in protein translation. Biochim. Biophys. Acta Gen. Subj..

[12] Cassidy L., Kaulich P.T., Maaß S., Bartel J., Becher D., Tholey A. (2021). Bottom-up and top-down proteomic approaches for the identification, characterization, and quantification of the low molecular weight proteome with focus on short open reading frame-encoded peptides. Proteomics.

[13] Rathore A. (2018).

[14] Delcourt V., Franck J., Quanico J., Gimeno J.P., Wisztorski M., Raffo-Romero A. (2018). Spatially-Resolved top-down proteomics bridged to MALDI MS imaging reveals the molecular physiome of brain regions. Mol. Cell Proteomics.

[15] Delcourt V., Franck J., Leblanc E., Narducci F., Robin Y.M., Gimeno J.P. (2017). Combined mass spectrometry imaging and top-down microproteomics reveals evidence of a hidden proteome in ovarian cancer. EBioMedicine.

[16] Saghatelian A., Couso J.P. (2015). Discovery and characterization of smORF-encoded bioactive polypeptides. Nat. Chem. Biol..

[17] Richardson M.O., Eddy S.R. (2023). ORFeus: a computational method to detect programmed ribosomal frameshifts and other non-canonical translation events. BMC Bioinformatics.

[18] Kochetov A.V., Allmer J., Kumar A. (2025).

[19] van Heesch S., Witte F., Schneider-Lunitz V., Schulz J.F., Adami E., Faber A.B. (2019). The translational landscape of the human heart. Cell.

[20] Wright B.W., Yi Z., Weissman J.S., Chen J. (2022). The dark proteome: translation from noncanonical open reading frames. Trends Cell Biol..

[21] Malekos E., Carpenter S. (2022). Short open reading frame genes in innate immunity: from discovery to characterization. Trends Immunol..

[22] Leong A.Z.-X., Lee P.Y., Mohtar M.A., Syafruddin S.E., Pung Y.-F., Low T.Y. (2022). Short open reading frames (sORFs) and microproteins: an update on their identification and validation measures. J. Biomed. Sci..

[23] Ma J., Ward C.C., Jungreis I., Slavoff S.A., Schwaid A.G., Neveu J. (2014). Discovery of human sORF-encoded polypeptides (SEPs) in cell lines and tissue. J. Proteome Res..

[24] Slavoff S.A., Mitchell A.J., Schwaid A.G., Cabili M.N., Ma J., Levin J.Z. (2013). Peptidomic discovery of short open reading frame–encoded peptides in human cells. Nat. Chem. Biol..

[25] Deutsch E.W., Kok L.W., Mudge J.M., Ruiz-Orera J., Fierro-Monti I., Sun Z. (2024). High-quality peptide evidence for annotating non-canonical open reading frames as human proteins. bioRxiv.

[26] Vanderperre B., Lucier J.F., Roucou X. (2012). HAltORF: a database of predicted out-of-frame alternative open reading frames in human. Database (Oxford).

[27] Brunet M.A., Brunelle M., Lucier J.-F., Delcourt V., Levesque M., Grenier F. (2019). OpenProt: a more comprehensive guide to explore eukaryotic coding potential and proteomes. Nucleic Acids Res..

[28] Buur L.M., Declercq A., Strobl M., Bouwmeester R., Degroeve S., Martens L. (2024). MS2Rescore 3.0 is a modular, flexible, and user-friendly platform to boost peptide identifications, as showcased with MS Amanda 3.0. J. Proteome Res..

[29] Gessulat S., Schmidt T., Zolg D.P., Samaras P., Schnatbaum K., Zerweck J. (2019). Prosit: proteome-wide prediction of peptide tandem mass spectra by deep learning. Nat. Methods.

[30] Degroeve S., Martens L. (2013). MS2PIP: a tool for MS/MS peak intensity prediction. Bioinformatics.

[31] Käll L., Canterbury J.D., Weston J., Noble W.S., MacCoss M.J. (2007). Semi-supervised learning for peptide identification from shotgun proteomics datasets. Nat. Methods.

[32] Mudge J.M., Ruiz-Orera J., Prensner J.R., Brunet M.A., Calvet F., Jungreis I. (2022). Standardized annotation of translated open reading frames. Nat. Biotechnol..

[33] Leblanc S., Yala F., Provencher N., Lucier J.F., Levesque M., Lapointe X. (2024). OpenProt 2.0 builds a path to the functional characterization of alternative proteins. Nucleic Acids Res..

[34] Brar G.A., Weissman J.S. (2015). Ribosome profiling reveals the what, when, where and how of protein synthesis. Nat. Rev. Mol. Cell Biol..

[35] Cardon T., Fournier I., Salzet M. (2021). Unveiling a ghost proteome in the glioblastoma non-coding RNAs. Front. Cell Developmental Biol..

[36] Cao X., Slavoff S.A. (2020). Non-AUG start codons: expanding and regulating the small and alternative ORFeome. Exp. Cell Res..

[37] Ivanov I.P., Firth A.E., Michel A.M., Atkins J.F., Baranov P.V. (2011). Identification of evolutionarily conserved non-AUG-initiated N-terminal extensions in human coding sequences. Nucleic Acids Res..

[38] Mohsen J.J., Martel A.A., Slavoff S.A. (2023). Microproteins—Discovery, structure, and function. Proteomics.

[39] Calvo S.E., Pagliarini D.J., Mootha V.K. (2009). Upstream open reading frames cause widespread reduction of protein expression and are polymorphic among humans. Proc. Natl. Acad. Sci. U. S. A..

[40] Lee S., Liu B., Lee S., Huang S.-X., Shen B., Qian S.-B. (2012). Global mapping of translation initiation sites in mammalian cells at single-nucleotide resolution. Proc. Natl. Acad. Sci. U. S. A..

[41] Huang J.-Z., Chen M., Chen D., Gao X.-C., Zhu S., Huang H. (2017). A peptide encoded by a putative lncRNA HOXB-AS3 suppresses colon cancer growth. Mol. Cell.

[42] Nichols C., Peltier D.C. (2024). Noncanonical microprotein regulation of immunity. Mol. Ther..

[43] Hofman D.A., Prensner J.R., van Heesch S. (2024). Microproteins in cancer: identification, biological functions, and clinical implications. Trends Genet..

[44] Khitun A., Ness T.J., Slavoff S.A. (2019). Small open reading frames and cellular stress responses. Mol. Omics.

[45] Martinez T.F., Lyons-Abbott S., Bookout A.L., De Souza E.V., Donaldson C., Vaughan J.M. (2023). Profiling mouse brown and white adipocytes to identify metabolically relevant small ORFs and functional microproteins. Cell Metab..

[46] Walters K., Guiterrez R., Sakhar S., Baldwin A., Nakayasu E., Russ H. (2024). 8681 proteogenomic identification of novel translated and regulatory open reading frames in human beta-cells. J. Endocr. Soc..

[47] Dagar S., Sharma M., Tsaprailis G., Tapia C.S., Crynen G., Joshi P.S. (2024). Ribosome profiling and mass spectrometry reveal widespread mitochondrial translation defects in a striatal cell model of Huntington disease. Mol. Cell Proteomics.

[48] Capuz A., Osien S., Karnoub M.A., Aboulouard S., Laurent E., Coyaud E. (2023). Astrocytes express aberrant immunoglobulins as putative gatekeeper of astrocyte-to-neuronal progenitor conversion. Cell Death Dis..

[49] Capuz A., Osien S., Cardon T., Karnoub M.A., Aboulouard S., Raffo-Romero A. (2023). Heimdall, an alternative protein issued from an ncRNA related to kappa light chain variable region of immunoglobulins in astrocytes: a new player in neural proteome. Cell Death Dis..

[50] Capuz A., Karnoub M.-A., Osien S., Rose M., Mériaux C., Fournier I. (2022). The antibody-dependent neurite outgrowth modulation response involvement in spinal cord injury. Front. Immunol..

[51] Cardon T., Franck J., Coyaud E., Laurent E.M., Damato M., Maffia M. (2020). Alternative proteins are functional regulators in cell reprogramming by PKA activation. Nucleic Acids Res..

[52] Duhamel M., Drelich L., Wisztorski M., Aboulouard S., Gimeno J.-P., Ogrinc N. (2022). Spatial analysis of the glioblastoma proteome reveals specific molecular signatures and markers of survival. Nat. Commun..

[53] Delcourt V., Brunelle M., Roy A.V., Jacques J.F., Salzet M., Fournier I. (2018). The protein coded by a short open reading frame, not by the annotated coding sequence, is the main gene product of the dual-coding gene MIEF1. Mol. Cell Proteomics.

[54] Cao X., Khitun A., Na Z., Dumitrescu D.G., Kubica M., Olatunji E. (2020). Comparative proteomic profiling of unannotated microproteins and alternative proteins in human cell lines. J. Proteome Res..

[55] Lee C., Kim K.H., Cohen P. (2016). MOTS-c: a novel mitochondrial-derived peptide regulating muscle and fat metabolism. Free Radic. Biol. Med..

[56] Chen Y., Su H., Zhao J., Na Z., Jiang K., Bacchiocchi A. (2023). Unannotated microprotein EMBOW regulates the interactome and chromatin and mitotic functions of WDR5. Cell Rep..

[57] Bryant P., Pozzati G., Elofsson A. (2022). Improved prediction of protein–protein interactions using AlphaFold2. Nat. Commun..

[58] Jumper J., Evans R., Pritzel A., Green T., Figurnov M., Ronneberger O. (2021). Highly accurate protein structure prediction with AlphaFold. Nature.

[59] Garcia-Del Rio D.F., Derhourhi M., Bonnefond A., Leblanc S., Guilloy N., Roucou X. (2024). Deciphering the ghost proteome in ovarian cancer cells by deep proteogenomic characterization. Cell Death Dis..

[60] Garcia-Del Rio D.F., Fournier I., Cardon T., Salzet M. (2023). Protocol to identify human subcellular alternative protein interactions using cross-linking mass spectrometry. STAR Protoc..

[61] Garcia-Del Rio D.F., Cardon T., Eyckerman S., Fournier I., Bonnefond A., Gevaert K. (2023). Employing a non-targeted interactomics approach and subcellular fractionation to increase our understanding of the ghost proteome. iScience.

[62] Cardon T., Hervé F., Delcourt V., Roucou X., Salzet M., Franck J. (2020). Optimized sample preparation workflow for improved identification of ghost proteins. Anal. Chem..

[63] Chen Y., Ho L., Tergaonkar V. (2021). sORF-Encoded MicroPeptides: new players in inflammation, metabolism, and precision medicine. Cancer Lett..

[64] Merino-Valverde I., Greco E., Abad M. (2020). The microproteome of cancer: from invisibility to relevance. Exp. Cell Res..

[65] Stein C.S., Jadiya P., Zhang X., McLendon J.M., Abouassaly G.M., Witmer N.H. (2018). Mitoregulin: a lncRNA-encoded microprotein that supports mitochondrial supercomplexes and respiratory efficiency. Cell Rep..

[66] Coellar J.D., Long J., Danesh F.R. (2021). Long noncoding RNAs and their therapeutic promise in diabetic nephropathy. Nephron.

[67] Li M., Shao F., Qian Q., Yu W., Zhang Z., Chen B. (2021). A putative long noncoding RNA-encoded micropeptide maintains cellular homeostasis in pancreatic β cells. Mol. Ther. Nucleic Acids.

[68] Baskin K.K., Makarewich C.A., DeLeon S.M., Ye W., Chen B., Beetz N. (2017). MED12 regulates a transcriptional network of calcium-handling genes in the heart. JCI Insight.

[69] Makarewich C.A., Olson E.N. (2017). Mining for micropeptides. Trends Cell Biol..

[70] Lanfranchi M., Yandiev S., Meyer-Dilhet G., Ellouze S., Kerkhofs M., Dos Reis R. (2024). The AMPK-related kinase NUAK1 controls cortical axon branching by locally modulating mitochondrial metabolic functions. Nat. Commun..

[71] Xiao W., Halabi R., Lin C.-H., Nazim M., Yeom K.-H., Black D.L. (2024). The lncRNA Malat1 is trafficked to the cytoplasm as a localized mRNA encoding a small peptide in neurons. Genes Development.

[72] Yang Y., Chen C., Li K., Zhang Y., Chen L., Shi J. (2025). Proteogenomic profiling reveals small ORFs and functional microproteins in activated T cells. Mol. Cell Proteomics.

[73] Bazzini A.A., Johnstone T.G., Christiano R., Mackowiak S.D., Obermayer B., Fleming E.S. (2014). Identification of small ORFs in vertebrates using ribosome footprinting and evolutionary conservation. EMBO J..

[74] Vakirlis N., Vance Z., Duggan K.M., McLysaght A. (2022). De novo birth of functional microproteins in the human lineage. Cell Rep..

[75] Vakirlis N., Hebert A.S., Opulente D.A., Achaz G., Hittinger C.T., Fischer G. (2018). A molecular portrait of de novo genes in yeasts. Mol. Biol. Evol..

[76] Ehx G., Larouche J.-D., Durette C., Laverdure J.-P., Hesnard L., Vincent K. (2021). Atypical acute myeloid leukemia-specific transcripts generate shared and immunogenic MHC class I-associated epitopes. Immunity.

[77] Choi M., Kang K.W. (2023). Mitoregulin controls mitochondrial function and stress-adaptation response during early phase of endoplasmic reticulum stress in breast cancer cells. Biochim. Biophys. Acta Mol. Basis Dis..

[78] Na Z., Luo Y., Schofield J.A., Smelyansky S., Khitun A., Muthukumar S. (2020). The NBDY microprotein regulates cellular RNA decapping. Biochemistry.

[79] Frion J., Meller A., Marbach G., Lévesque D., Roucou X., Boisvert F.-M. (2023). CRISPR/Cas9-mediated knockout of the ubiquitin variant UbKEKS reveals a role in regulating nucleolar structures and composition. Biol. Open.

[80] Brunet M.A., Leblanc S., Roucou X. (2020). Reconsidering proteomic diversity with functional investigation of small ORFs and alternative ORFs. Exp. Cell Res..

[81] Menschaert G., Van Criekinge W., Notelaers T., Koch A., Crappé J., Gevaert K. (2013). Deep proteome coverage based on ribosome profiling aids mass spectrometry-based protein and peptide discovery and provides evidence of alternative translation products and near-cognate translation initiation events. Mol. Cell Proteomics.

[82] Kaulich P.T., Cassidy L., Bartel J.R., Schmitz R.A., Tholey A. (2021). Multi-protease approach for the improved identification and molecular characterization of small proteins and short open reading frame-encoded peptides. J. Proteome Res..

[83] Cassidy L., Kaulich P.T., Tholey A. (2023). Proteoforms expand the world of microproteins and short open reading frame-encoded peptides. iScience.

[84] Cassidy L., Kaulich P.T., Tholey A. (2019). Depletion of high-molecular-mass proteins for the identification of small proteins and short open reading frame encoded peptides in cellular proteomes. J. Proteome Res..

[85] Pueyo J.I., Magny E.G., Sampson C.J., Amin U., Evans I.R., Bishop S.A. (2016). Hemotin, a regulator of phagocytosis encoded by a small ORF and conserved across metazoans. PLoS Biol..

[86] Baena-Angulo C., Platero A.I., Couso J.P. (2024). Cis to trans: small ORF functions emerging through evolution. Trends Genet..

[87] Zhao B., Zhao J., Wang M., Guo Y., Mehmood A., Wang W. (2023). Exploring microproteins from various model organisms using the MiP-mining database. BMC Genomics.

[88] D’Lima N.G., Ma J., Winkler L., Chu Q., Loh K.H., Corpuz E.O. (2017). A human microprotein that interacts with the mRNA decapping complex. Nat. Chem. Biol..

[89] Chambers M.C., Jagtap P.D., Johnson J.E., McGowan T., Kumar P., Onsongo G. (2017). An accessible proteogenomics informatics resource for cancer researchers. Cancer Res..

[90] Miller B.F., Hamilton K.L. (2017). Overview: the modulation of ageing through altered proteostasis. J. Physiol..

[91] Nishimoto I., Matsuoka M., Niikura T. (2004). Unravelling the role of Humanin. Trends Mol. Med..

[92] Lee C., Zeng J., Drew B.G., Sallam T., Martin-Montalvo A., Wan J. (2015). The mitochondrial-derived peptide MOTS-c promotes metabolic homeostasis and reduces obesity and insulin resistance. Cell Metab..

[93] Mittal N., Ataman M., Tintignac L., Ham D.J., Jörin L., Schmidt A. (2024). Calorie restriction and rapamycin distinctly restore non-canonical ORF translation in the muscles of aging mice. NPJ Regenerative Med..

[94] Fesenko I., Sahakyan H., Dhyani R., Shabalina S.A., Storz G., Koonin E.V. (2025). The hidden bacterial microproteome. Mol. Cell.

[95] Sberro H., Fremin B.J., Zlitni S., Edfors F., Greenfield N., Snyder M.P. (2019). Large-scale analyses of human microbiomes reveal thousands of small, novel genes. Cell.

[96] Makarewich C.A., Munir A.Z., Schiattarella G.G., Bezprozvannaya S., Raguimova O.N., Cho E.E. (2018). The DWORF micropeptide enhances contractility and prevents heart failure in a mouse model of dilated cardiomyopathy. eLife.

[97] Giacomini G., Graziani C., Lachi V., Bongini P., Pancino N., Bianchini M. (2022). A neural network approach for the analysis of reproducible Ribo–Seq profiles. Algorithms.

[98] Limbu M.S., Xiong T., Wang S. (2024). A review of ribosome profiling and tools used in Ribo-seq data analysis. Comput. Struct. Biotechnol. J..

[99] Jacob A.G., Smith C.W. (2017). Intron retention as a component of regulated gene expression programs. Hum. Genet..

[100] Su H., Katz S.G., Slavoff S.A. (2024). Alternative transcripts recode human genes to express overlapping, frameshifted microproteins. bioRxiv.

[101] Bogard B., Francastel C., Hubé F. (2020). Multiple information carried by RNAs: total eclipse or a light at the end of the tunnel?. RNA Biol..

[102] Monteuuis G., Wong J.J., Bailey C.G., Schmitz U., Rasko J.E. (2019). The changing paradigm of intron retention: regulation, ramifications and recipes. Nucleic Acids Res..

[103] Dong C., Cesarano A., Bombaci G., Reiter J.L., Yu C.Y., Wang Y. (2021). Intron retention-induced neoantigen load correlates with unfavorable prognosis in multiple myeloma. Oncogene.

[104] Dong C., Reiter J.L., Dong E., Wang Y., Lee K.P., Lu X. (2022). Intron-retention neoantigen load predicts favorable prognosis in pancreatic cancer. JCO Clin. Cancer Inform..

[105] Park J., Chung Y.-J. (2019). Identification of neoantigens derived from alternative splicing and RNA modification. Genomics Inform..

[106] Pyke R.M., Mellacheruvu D., Dea S., Abbott C., Zhang S.V., Phillips N.A. (2023). Precision neoantigen discovery using large-scale immunopeptidomes and composite modeling of MHC peptide presentation. Mol. Cell Proteomics.

[107] Purcell A.W., Gorman J.J. (2004). Immunoproteomics: mass spectrometry-based methods to study the targets of the immune response. Mol. Cell Proteomics.

[108] Anderson D.M., Anderson K.M., Chang C.L., Makarewich C.A., Nelson B.R., McAnally J.R. (2015). A micropeptide encoded by a putative long noncoding RNA regulates muscle performance. Cell.

